# Verapamil Attenuated Prediabetic Neuropathy in High-Fat Diet-Fed Mice through Inhibiting TXNIP-Mediated Apoptosis and Inflammation

**DOI:** 10.1155/2019/1896041

**Published:** 2019-01-10

**Authors:** Lingling Xu, Xiaopu Lin, Meiping Guan, Yanmei Zeng, Yingshan Liu

**Affiliations:** ^1^Department of Endocrinology, Shenzhen Hospital, Southern Medical University, China; ^2^Department of Huiqiao Building, Nanfang Hospital, Southern Medical University, China; ^3^Department of Endocrinology, Nanfang Hospital, Southern Medical University, China

## Abstract

Diabetic neuropathy (DN) is a common and severe complication of diabetes mellitus. There is still a lack of an effective treatment to DN because of its complex pathogenesis. Thioredoxin-interacting protein (TXNIP), an endogenous inhibitor of thioredoxin, has been shown to be associated with diabetic retinopathy and nephropathy. Herein, we aim to investigate the role of TXNIP in prediabetic neuropathy and therapeutic potential of verapamil which has been shown to inhibit TXNIP expression. The effects of mediating TXNIP on prediabetic neuropathy and its exact mechanism were performed using high-fat diet- (HFD-) induced diabetic mice and palmitate-treated neurons. Our results showed that TXNIP upregulation is associated with prediabetic neuropathy in HFD-fed mice. TXNIP knockdown improved DN in HFD-induced prediabetic mice. Mechanistically, increased TXNIP in dorsal root ganglion is transferred into the cytoplasm and shuttled to the mitochondria. In cytoplasm, TXNIP binding to TRX1 results in the increased oxidative stress and inflammation. In mitochondria, TXNIP binding to TRX2 induced mitochondria dysfunction and apoptosis. TXNIP isolated from TRX2 then shuttles to the cytoplasm and binds to NLRP3, resulting in further increased TXNIP-NLRP3 complex, which induced the release of IL-1*β* and the development of inflammation. Thus, apoptosis and inflammation of dorsal root ganglion neuron eventually cause neural dysfunction. In addition, we also showed that verapamil, a known inhibitor of calcium channels, improved prediabetic neuropathy in the HFD-fed mice by inhibiting the upregulation of TXNIP. Our finding suggests that TXNIP might be a potential target for the treatment of neuropathy in prediabetic patients with dyslipidemia.

## 1. Introduction

Diabetic neuropathy (DN) is a common and severe complication of diabetes mellitus and affects nearly 20% of adult diabetic patients [1]. DN is associated with pain, decreased motility, and amputation, which significantly reduce the life quality of patients [2]. Because of its complex pathogenesis, few available agents provide the effective improvements in diabetic neuropathy with the exception of good glycemic control [3]. Thus, earlier intervention might be more important for delaying the progression of DN.

Preclinical and clinical data demonstrated that the metabolic syndrome associated with obesity is a risk factor for the occurrence of peripheral neuropathy [4–6]. In the prediabetes, abnormal lipid metabolism leads to the occurrence of peripheral neuropathy [7, 8]. Our previous study confirmed that high-fat diet (HFD) induced peripheral neuropathy in C57BL/6 mice before the onset of diabetes, which may be related to oxidative stress [9]. However, the exact molecular mechanism is still not clear.

Thioredoxin-interacting protein (TXNIP), also known as thioredoxin-binding protein-2 (TBP-2), is an endogenous inhibitor of thioredoxin (TRX) that together with glutathione regulates the oxidative stress in cells facing various stress [10]. TXNIP plays an essential role in diverse biological processes, including regulation of oxidative stress, inflammation, glucose and lipid metabolism, and cell apoptosis [11]. TXNIP overexpression causes an unbalance of these biological processes, which have been known as important contributors to the emergence of multiple diabetic complications, including diabetic retinopathy [12, 13] and diabetic nephropathy [14, 15]. However, whether TXNIP overexpression is involved in the onset and progression of DN remains unknown, especially diabetic patients with dyslipidemia.

TXNIP expression is robustly induced by glucose [16, 17]. Extracellular glucose induces TXNIP expression through increased glycolytic intermediates, which are inducers of ChREBP/MondoA–Mlx transcription factors binding to carbohydrate response element (ChRE) in the TXNIP promoter [18, 19]. Carbohydrate response element-binding protein (ChREBP) has been identified as a key contributor to fatty acid synthesis under physiological and pathological conditions. For instance, HFD-fed mice overexpressing ChREBP showed greater hepatic steatosis [20]. In a recent study, HFD-fed mice showed an increased expression of ChREBP and TXNIP, which is associated with HFD-induced nonalcoholic fatty liver disease (NAFLD) [21]. Specifically, Price et al. reported that TXNIP is significantly increased in sensory neurons in the diabetic rats [22]. Thus, we hypothesized that TXNIP overexpression involved in the development and progression of DN in the subject with dyslipidemia. Verapamil, an inhibitor of calcium channels, is widely used for the treatment of high blood pressure [23]. Verapamil was first identified to reduce the cardiac expression of TXNIP *in vivo* in 2009 [24]. Furthermore, verapamil was shown to decrease *β*-cell TXNIP expression through inhibiting carbohydrate response element-binding protein (ChREBP) binding to the TXNIP promoter, which promoted *β*-cell survival and improved glucose homeostasis in BTBR ob/ob mice and STZ-induced mice [25].

The present studies were therefore aimed at determining whether TXNIP is associated with the development of DN and verapamil may enhance the survival of neurons and improve DN through decreasing TXNIP expression. Using HFD-induced diabetic mice and palmitate-treated neurons, we showed that oral verapamil reduced the expression of TXNIP, inhibited the activation of inflammasome, prevented the neuronal apoptotic, and eventually improved diabetic neuropathy. Thus, we show for the first time that TXNIP might be a potential target for the treatment of diabetic neuropathy.

## 2. Methods

### 2.1. Preparation of High-Fat Diet-Fed Mice

Three-week-old, female, C57/BL6 mice were obtained from the Laboratory Animal Center of Academy of Military Medical Sciences (Beijing, China). All animal procedures were conformed to the standard procedures approved by the Institutional Animal Ethics Committee of Southern Medical University. The mice were housed in a temperature-controlled environment (22 ± 1°C) with free access to water and food. After adaptive feeding for a week, the mice were randomly assigned to four groups as follows: normal control mice (control) with a placebo, high-fat diet-fed mice (HF) with a placebo, HF mice treated with verapamil (HF + verapamil), and control mice treated with verapamil (verapamil) (*n* = 10). The HF diet was composed of 35% carbohydrate, 45% fat, and 20% protein by energy (#D12451, Research Diets, New Brunswick, NJ, USA) that contain lard and soybean oil as fat sources, while the control diet contained 70% carbohydrate, 10% fat, and 20% protein (#D12450B, Research Diets) [26, 27]. After being fed with an HFD, mice were intragastrically administered with verapamil (St. Louis, MO, USA) 10 mg/kg dose once a day. According to the previous report showing that neuropathy and impaired glucose tolerance appeared after 34 weeks on high-fat diet [6], verapamil administration would be halted at the 34th week after mice were fed with an HFD.

The mice from each group were weighed at 8, 16, and 34 weeks, respectively. Heart rate, systolic blood pressure, diastolic blood pressure, and mean blood pressure were measured at 8, 16, and 34 weeks using CODA® HT System with 6 activated channels (Kent Scientific, Torrington, CT, USA). Briefly, all mice were acclimated by following a 7-day training protocol prior to the actual measurement. During blood measurement, mice were restrained in the warm bags stable at around 35–37°C. According to the manufacturer's protocol, heart rate and blood pressure were continuously recorded using volume pressure recording (VPR) sensor technology. At least 10 successive measurements were taken to calculate the averaged values.

### 2.2. Glucose Tolerance Tests

For fasting the intraperitoneal glucose tolerance test (ipGTT), mice were fed and then fasted for 6 h. After that, glucose (1 g/kg body weight) was injected intraperitoneally and blood was collected at 30 min, 60 min, and 120 min from the tail vein. Plasma glucose was measured using LifeScan OneTouch test paper on a SureStep glucose kit (Johnson & Johnson Company, CA, USA). The area under the curve (AUC) of the plasma glucose response was calculated as an index of glucose tolerance through trapezoidal analysis [28].

### 2.3. Analysis of Serum Lipid Profiles

All the mice were fasted for 12 h and killed with an overdose of pentobarbital when HF mice exhibited impaired glucose tolerance (IGT) for the first time (at the 34th week). Then, the blood samples were collected by removing eyeball and serums were isolated by centrifuging at 2000*g* for 10 min. Serum concentrations of triglycerides (TGs), total cholesterol (TC), HDL cholesterol (HDL-C), and LDL cholesterol (LDL-C) were determined with assay kits according to the manufacturer's directions (Sigma-Aldrich) on a Hitachi 7600 automatic biochemical analyzer (Hitachi Co., Tokyo, Japan).

### 2.4. Nerve Conduction Velocity (NCV) Studies

Mice were anesthetized with ketamine (30 mg/kg)/acepromazine (10 mg/kg) by peritoneal injection after 34 weeks of being fed with HFD when HF mice exhibited IGT. Following the anesthetization, the NCV was measured using a PowerLab/8 s data recording system (AD Instruments, Castle Hill, Australia) with the previous described methods [29]. The motor NCV (MNCV) and the sensory NCV (SNCV) were calculated by dividing the distance between stimulating and recording electrodes by the latency time difference. The measurement was performed for 3 times with an interval of 15 min.

### 2.5. Western Blot Analysis

The sciatic nerve proteins were extracted using T-PER tissue protein extraction reagent, and the neuron cells were lysed in M-PER mammalian protein extraction reagent, while the nuclear/cytosolic and mitochondria extracts were prepared using nuclear/cytoplasmic extraction kit and mitochondria isolation kit (Thermo Fisher Scientific, Waltham, MA, USA), respectively. The protein concentrations were determined by a BCA protein assay kit (Thermo Fisher Scientific), and equal amounts of sciatic nerve proteins or cell lysates were subjected to SDS-PAGE and then transferred to PVDF membrane (Millipore, Billerica, MA, USA). After blocking with 5% milk, the membranes were incubated with antibodies against TXNIP, NLRP3, caspase-1, IL-1*β*, GAPDH, TRX1, TRX2, and ASK1 (Cell Signaling Technology, Beverly, MA, USA), respectively, followed by incubating with the HRP-conjugated secondary antibodies. Lastly, chemiluminescence was detected using an ECL kit (GE Healthcare Life Sciences, Pittsburgh, PA, USA).

### 2.6. DRG Neuron Cultivation and Treatment of DRG Neurons with Palmitate-BSA

Dorsal root ganglions (DRGs) were collected from two anesthetized seven-week-old mice and were cultured as described previously [30]. Briefly, the DRGs were harvested and dissociated in 0.2% collagenase for 30 min followed by 1% trypsin for 15 min. DRG neurons were cultured in DMEM/Ham's F-12, 50/50 (Corning), containing 50 ng/mL NGF (Invitrogen), 2% B-27 (Invitrogen), 40 *μ*mol/L 5-fluorodeoxyuridine, and 1% antibiotic-antimycotic. On day 3, the medium was replaced with fresh medium not containing 5-fluorodeoxyuridine.

Palmitate, a saturated nonesterified fatty acid (NEFA), has been shown to induce ER stress [31], which is associated with increased TXNIP expression [32]. We thus used palmitate-BSA to increase TXNIP expression in the DRGs. Palmitate-BSA was made according to the published protocol [33]. The neurons were then treated with or without palmitate-BSA in the presence or absence of verapamil. The neurons were divided into seven groups: normal control cells (control), cells cultivated under the condition containing 600 *μ*M palmitate-BSA (HF), HF cells treated with 50 *μ*M verapamil (HF + VP (50 *μ*M)), HF cells treated with 100 *μ*M verapamil (HF + VP (100 *μ*M)), HF cells treated with 150 *μ*M verapamil (HF + VP (150 *μ*M)), HF cells transfected with 50 nM scrambled siRNA (HF + NC), and HF cells transfected with 50 nM TXNIP-siRNA (HF + TXNIP-siRNA). The culture supernatant was collected, and the IL-1*β* level was estimated using a mouse IL-1*β* ELISA kit (R&D Systems, Minneapolis, MN, USA).

### 2.7. Measurement of Mitochondrial Transmembrane Potential

Mitochondrial transmembrane potential (Δ*ψ*m) was assessed using a JC-1 mitochondrial membrane potential assay kit (Nanjing Jiancheng Bioengineering Institute, China). Briefly, after indicated treatments, the cells were incubated with an equal volume of JC-1 staining solution (5 *μ*g/mL) at 37°C for 20 min, then washed twice with PBS, and placed in fresh medium. Mitochondrial membrane potentials were monitored using a Beckman Coulter Altra flow cytometer (Beckman Coulter, Fullerton, CA, USA) with a 488 nm argon laser. In healthy cells, the JC-1 accumulates in the mitochondrial to form J-aggregates, which become red fluorescence. In apoptotic cells, the JC-1 cannot accumulate within the mitochondria and remains in the cytoplasm in a monomeric form to show green fluorescence. The intensity of green fluorescence delegates J-monomers which is quantified in FL-1 (*X*-axis), while the intensity of red fluorescence delegates J-aggregates which is quantified in FL-2 (*Y*-axis).

### 2.8. Cell Viability Assay

Cell viability was assessed by MTT assay. Cells (1 × 10^4^ per well) were seeded into 96-well culture plates and routinely cultured for 48 h. Then, 20 *μ*L of 3-(4,5-dimethylthiazol-2-yl)-2,5-diphenyltetrazolium bromide (MTT) (5 mg/mL) (Promega, Madison, WI, USA) was added and incubated at 37°C for another 4 h, followed by solubilization in 150 *μ*L dimethyl sulfoxide (DMSO). The wavelength absorption values were measured at 570 nm on Enspire (Perkin Elmer, Waltham, MA, USA).

### 2.9. Caspase-3 Activity Assay

Caspase-3 activity was measured by cleavage of chromogenic caspase substrates (Ac-DEVD-pNA) (Beyotime). Approximately 50 *μ*g of total protein of cell lysate was added to reaction buffer containing 2 mM Ac-DEVD-pNA and incubated at 37°C for 2 h, and then, the absorbance of pNA cleavage was determined at 405 nm on Enspire.

### 2.10. Immunofluorescence Assay

Neuron cells grown on glass coverslips were treated with 600 *μ*M palmitate-BSA for 24 h, 48 h, and 72 h, respectively. After treatment, the culture medium was removed and the cells were washed and fixed with 4% formaldehyde. After that, the cells were permeabilized in 0.5% Triton X-100 for 15 min and blocked by 1% BSA for 1 h at room temperature. Cells were then incubated with a rabbit anti-TXNIP antibody for 2 h and incubated with FITC-conjugated goat anti-rabbit secondary antibody (Jackson ImmunoResearch, West Grove, PA, USA) for 1 h. Lastly, Hoechst (Beyotime) was added for 10 min and images were obtained using a fluorescence microscope (Leica, Wetzlar, Germany).

### 2.11. Coimmunoprecipitation Analysis

Neuron cells were cultured in a 6-well plate to 80% confluency. Cytosolic and mitochondria extracts were prepared using a mitochondria extraction kit (Thermo Fisher Scientific). Briefly, cells were resuspended in lysis buffer and disrupted with a Dounce homogenizer. Then, homogenates were centrifuged at 700 ×*g* to pellet nuclei and cell debris. After centrifuging the supernatants at 12,000 ×*g*, the cytosolic fractions (supernatant) were collected. Next, the supernatant was further lysed by mitochondria isolation reagent. The mitochondria fractions were obtained after centrifuging at 12,000 ×*g* and discarding the supernatant. Thereafter, protein concentrations were quantified. Lysates containing 500 *μ*g protein were incubated with target antibodies (anti-TXNIP antibody, anti-TRX1 antibody, and anti-TRX2 antibody) or control IgG antibody (Cell Signaling Technology) at 4°C for 4 h. Then, 50 *μ*L of Protein A/G Sepharose (GE Healthcare) was added, followed by incubation at 4°C overnight. After incubation, beads were washed and resuspended in 50 *μ*L of 2 × SDS-PAGE sample buffer. The sample was heated and collected for western blot analysis.

### 2.12. Statistical Analyses

Statistical analyses were performed using the GraphPad Prism 6.0 software. Results are expressed as mean ± SEM or SD. Data were analyzed by one-way ANOVA with the Holm-Sidak multiple comparison test. *P* < 0.05 was considered significant.

## 3. Results

### 3.1. Verapamil Avoids Increases of Weight, Heart Rate, and Blood Pressure of HFD-Fed Mice

**Figure 1:**
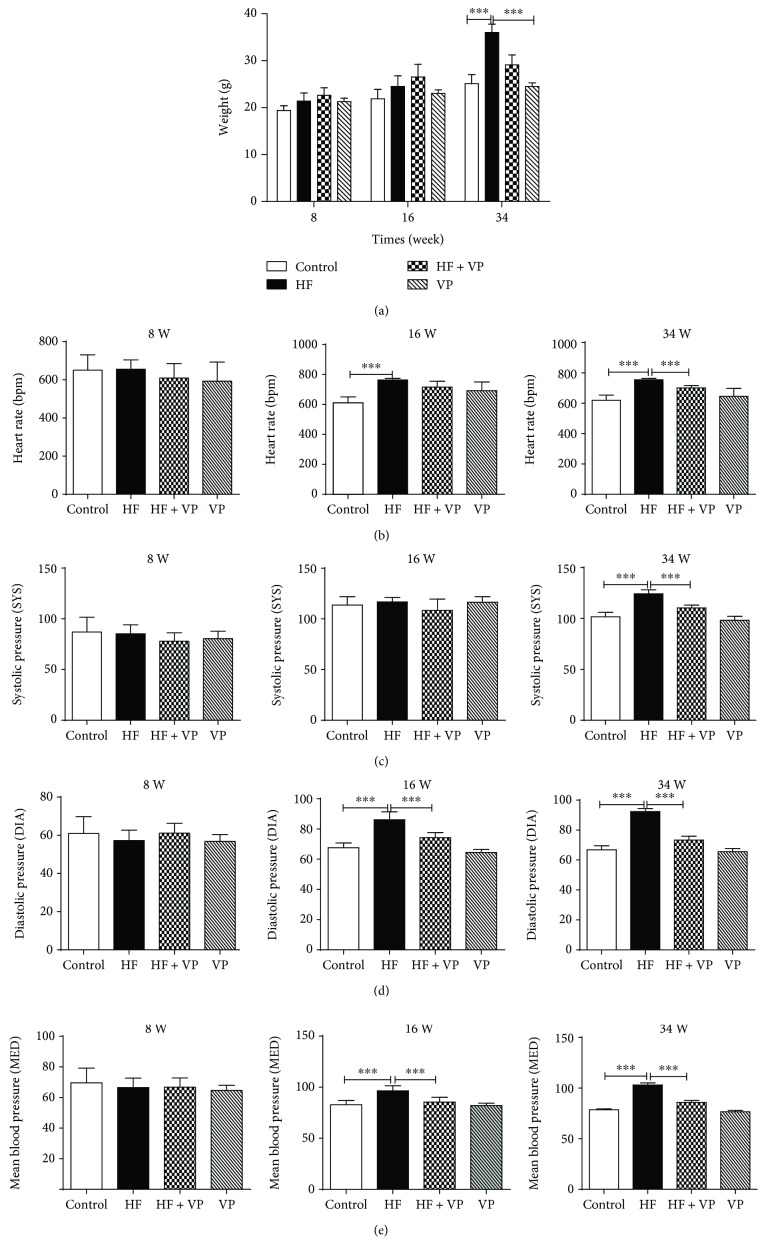
Effects of verapamil treatment on weight, heart rate, and blood pressure of high-fat diet-fed mice. C57/BL6 mice (*n* = 10) were fed with normal diet (control) or high-fat diet (HF). HF mice and control mice were treated with verapamil (indicated as HF + VP and VP). The body weight (a), heart ratio (b), diastolic pressure (c), systolic pressure (d), and mean blood pressure (e) of mice were measured at weeks 8, 16, and 34, respectively. Each value represents mean ± SEM (*n* = 10). ^∗∗∗^*P* < 0.001.

Following 34 weeks on an HFD, the body weight was increased in the HFD-fed mice (HF group) compared to the normal diet-fed mice (control group) at the initial first 8 weeks, and the weight gain continued to be progressively higher in HF mice, especially at week 34 (*P* < 0.001) (Figure 1(a)). Meanwhile, the heart rate of mice with HFD was also enhanced along with time. The enhancement was especially significant at weeks 16 and 34 (*P* < 0.001) (Figure 1(b)). Similar with body weight and heart ratio, the blood pressure including diastolic pressure, systolic pressure, and mean blood pressure of HF mice was markedly increased at weeks 16 and/or 34 (Figures 1(c)–1(e)), while verapamil, a calcium channel blocker, significantly inhibits increases of weight, heart rate, and blood pressure including diastolic pressure, systolic pressure, and mean blood pressure in HF mice, especially at weeks 16 and/or 34 (*P* < 0.001). Besides, there were no significant differences in terms of weight, heart rate, and blood pressure between the control group treated with verapamil (VP group) and the control group (Figures 1(a)–1(e)).

### 3.2. Verapamil Prevents IGT and Lowers Serum Lipids in HFD-Fed Mice

**Figure 2:**
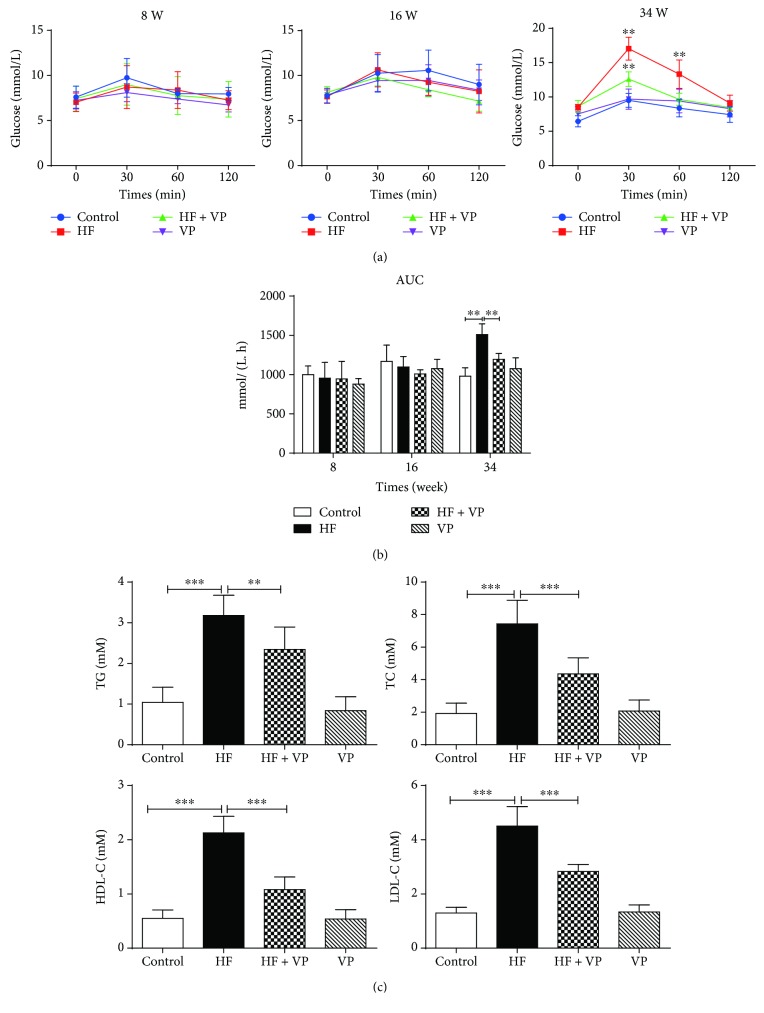
Effects of verapamil on glucose intolerance and serum lipid profiles of HF mice. (a) The curve of ipGTT was shown based on plasma glucose concentrations in the indicated time. At weeks 8, 16, and 34, mice were fasted and injected with glucose. The blood was collected at 0 min, 30 min, 60 min, and 120 min from the tail vein, and the blood glucose levels were measured. (b) The AUC of the plasma glucose response was then calculated according to ipGTT results. (c) The serum levels of TG, TC, HDL-C, and LDL-C were determined. In the end of a 34-week study, four groups of mice were fasted for 12 h, and the blood serums were collected and TG, TC, HDL-C, and LDL-C were measured. Each value represents mean ± SEM (*n* = 10). ^∗∗^*P* < 0.01; ^∗∗∗^*P* < 0.001.

A glucose tolerance test was performed at 8 weeks, 16 weeks, and 34 weeks after introduction of high-fat diet. The result showed that there were no obvious changes on glucose level of mice with HFD within 16 weeks, while HFD feeding over 34 weeks induced a significant increase of plasma glucose levels for mice compared with the control group. The HF mice displayed IGT with significantly increased blood glucose levels 30 min and 60 min after injecting glucose at 34 weeks (*P* < 0.01). Along the lines of reduced weight, heart rate, and blood pressure, treatment of verapamil decreased the plasma glucose concentrations of HF mice at indicated times (*P* < 0.01), while exhibiting no obvious effect on control mice. (Figure 2(a)). The AUC for plasma glucose, calculated as the change from basal glucose values in each subject, was also significantly reduced in verapamil-treated HF mice compared to HF mice at 34 weeks (*P* < 0.01) (Figure 2(b)).

Following the glucose tolerance test, we next evaluated the effects of verapamil on serum lipid profiles in HFD-fed mice. The serum concentrations of lipids including TG, TC, HDL-C, and LDL-C in mice with HFD were significantly increased compared with normal diet-fed mice (*P* < 0.001). However, treatment with verapamil significantly lowered serum TG, TC, HDL-C, and LDL-C concentrations (*P* < 0.01 or 0.001), as shown in Figure 2(c). On the other side, verapamil treatment showed no great changes on these indexes in control mice (Figure 2(c)).

### 3.3. Verapamil Partially Restores Nerve Conduction Velocity and Inhibits the Expression of TXNIP, NLRP3, Caspase-1, and IL-1*β* in HFD-Fed Mice

**Figure 3:**
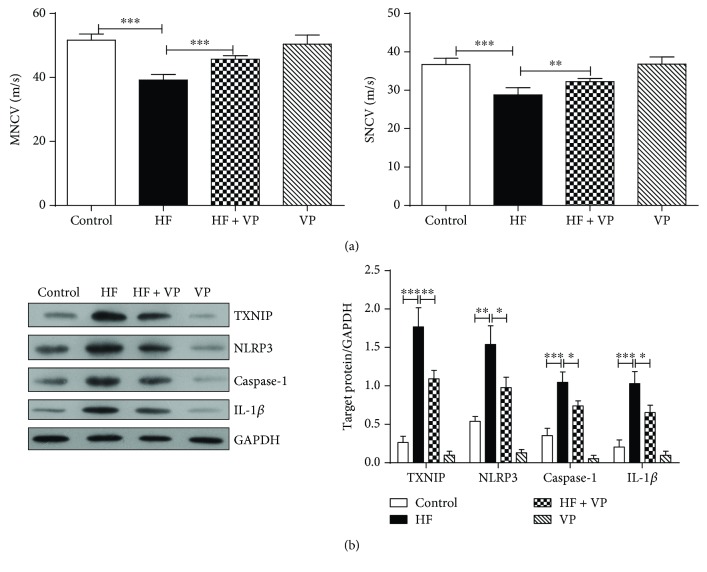
Effects of verapamil on nerve conduction velocity and protein expressions related to metabolism and inflammation in high-fat diet-fed mice. (a) The MNCV and SNCV were detected. After HF mice exhibited IGT for the first time, four groups of mice were anesthetized, and MNCV and SNCV were measured for 3 times with an interval of 15 min. Each value represents mean ± SEM (*n* = 10). ^∗∗^*P* < 0.01; ^∗∗∗^*P* < 0.001. (b) The expressions of TXNIP, NLRP3, caspase-1, and IL-1*β* were detected. The sciatic nerve was isolated, and the protein expressions in sciatic nerve including TXNIP, NLRP3, caspase-1, and IL-1*β* were determined by western blotting analysis, and the target protein expressions relative to GAPDH expression were displayed on the right. The experiments were performed in triplicate, and each value represents mean ± SD. ^∗^*P* < 0.05; ^∗∗^*P* < 0.01; ^∗∗∗^*P* < 0.001.

Following 34 weeks of HFD feeding and verapamil treatment, the MNCV and SNCV were assessed. Data in Figure 3(a) demonstrated that control mice treated with verapamil had no effect on MNCV and SNCV. MNCV and SNCV were significantly decreased in HFD-fed mice compared to control mice (*P* < 0.001), and they were significantly increased in HFD-fed mice treated with verapamil application (*P* < 0.01 or 0.001). Furthermore, the sciatic nerve was isolated and the expressions of proteins related to metabolism and inflammation including TXNIP, NLRP3, caspase-1, and IL-1*β* were determined. As indicated in Figure 3(b), the protein levels of TXNIP, NLRP3, caspase-1, and IL-1*β* were unchanged in control mice with verapamil treatment. Of note, their expressions were markedly elevated in HFD-fed mice without verapamil treatment while verapamil treatment obviously avoided the increases of their expressions.

### 3.4. TXNIP Inhibition Decreased the Expression of NLRP3, Caspase-1, and IL-1*β* in Palmitate-Treated Neurons

**Figure 4:**
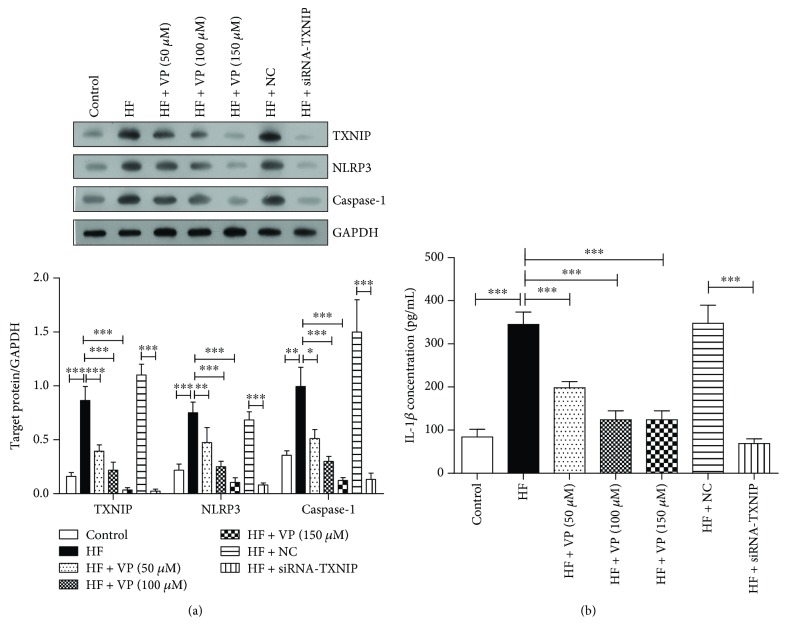
The effect of TXNIP inhibition on NLRP3, caspase-1, and IL-1*β* expressions in palmitate-treated neurons. Dorsal root ganglions were isolated from two seven-week-old normal diet-fed mice, and the neurons were cultivated and divided into seven groups: control, HF, HF + VP (50 *μ*M), HF + VP (100 *μ*M), HF + VP (150 *μ*M), HF + NC, and HF + TXNIP-siRNA. (a) The protein expressions of TXNIP, NLRP3, and caspase-1 were detected through western blotting assay. The target protein expressions relative to GAPDH expression were displayed below. The experiments were performed in triplicate, and each value represents mean ± SD. ^∗^*P* < 0.05; ^∗∗^*P* < 0.01; ^∗∗∗^*P* < 0.001. (b) The IL-1*β* level in supernatant was determined by ELISA assay. The experiments were performed in triplicate, and each value represents mean ± SD. ^∗∗∗^*P* < 0.001.

To determine the mechanism of verapamil, we first isolated the dorsal root ganglions from normal diet-fed mice and cultured neurons, and then we treated neurons with 600 *μ*M palmitate-BSA to provide the high-fat condition. Meanwhile, the cells were treated with different concentrations of verapamil, scrambled siRNA, and TXNIP-siRNA, respectively. Similar to the *in vivo* results, the data from Figure 4(a) indicated that high-fat treatment induced the expression of TXNIP, NLRP3, and caspase-1. However, verapamil inhibited the expression of TXNIP as well as the protein expression of NLRP3 and caspase-1 in palmitate-BSA-treated neurons dose dependently. On the other hand, TXNIP-siRNA also reduced TXNIP, NLRP3, and caspase-1 expression compared to scrambled siRNA. Furthermore, the result showed that verapamil and TXNIP-siRNA reduced IL-1*β* secretion in palmitate-BSA-treated neurons through ELISA assay (Figure 4(b)). Hence, verapamil, as a TXNIP inhibitor, decreased the expression of NLRP3, caspase-1, and IL-1*β* in high-fat-treated neurons.

### 3.5. TXNIP Inhibition Attenuated Cell Apoptosis and Promoted Cell Viability in Palmitate-Treated Neurons

**Figure 5:**
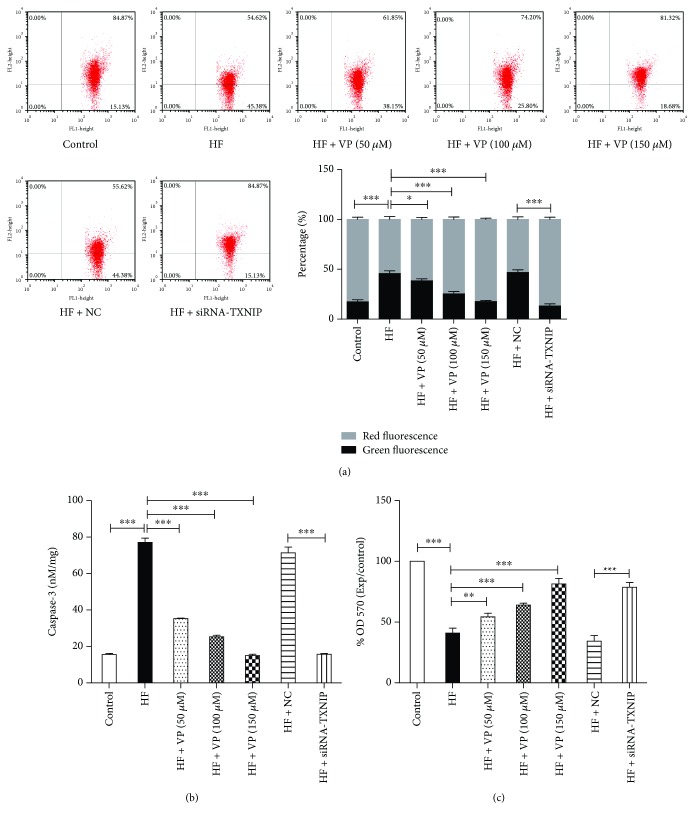
The effect of TXNIP inhibition on cell apoptosis and promoted cell viability in palmitate-treated neurons. (a) The changes in the Δ*ψ*m based on JC-1 staining were measured by a flow cytometer. The intensity of green fluorescence delegates JC-1 monomer which is quantified in FL-1 (*X*-axis), while the intensity of red fluorescence delegates J-aggregates which is quantified in FL-2 (*Y*-axis). The proportions of green fluorescence and red fluorescence were displayed on the right. The experiments were performed in triplicate. (b) The activity of caspase-3 was measured by pNA, was released from the caspase substrate Ac-DEVD-pNA, and was reported as OD 405 nM/mg protein. (c) The cell viability was measured by MTT assay. The experiments were performed in triplicate, and each value represents mean ± SD. ^∗^*P* < 0.05; ^∗∗^*P* < 0.01; ^∗∗∗^*P* < 0.001.

TXNIP-mediated IL-1*β* induction plays an essential role in the initiation of mitochondrial apoptosis; therefore, we next assessed the changes in Δ*ψ*m with a method based on JC-1 staining. Compared to the normal neurons with or without verapamil, we found that the palmitate-treated neurons showed a higher proportion of J-monomers with green fluorescence and lower proportion of J-aggregates with red fluorescence, suggesting that palmitate treatment initiated the induction of mitochondrial apoptosis (Figure 5(a)). In line with this, we observed significantly increased level of caspase-3 and reduced cell viability in palmitate-treated neurons compared to the normal neurons (Figures 5(b) and 5(c)). Moreover, our results showed that verapamil dose-dependently decreased the proportion of J-monomers and increased the proportion of J-aggregates in palmitate-treated cells. We also observed decreased activity of caspase-3 and the increased cell viability in the palmitate-treated neurons in the presence of verapamil. Similarly, siRNA-mediated inhibition of TXNIP significantly decreased the proportion of J-monomers and increased the proportion of J-aggregates and the expression of caspase-3 and increased the cell viability in palmitate-treated neurons (Figure 5). Taken together, these results suggest that TXNIP inhibition can attenuate the apoptosis of neurons induced by palmitate.

### 3.6. TXNIP Transfers from the Nucleus into Cytosol and Mitochondria to Mediate Oxidative Stress under High-Fat Condition

**Figure 6:**
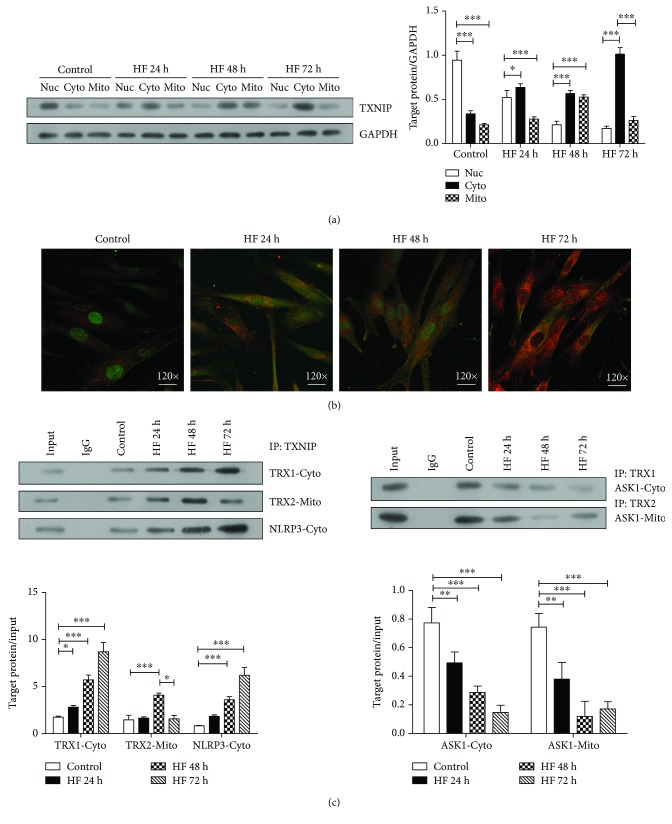
The distribution and location of TXNIP in palmitate-treated neurons. (a) The distribution of TXNIP in the nucleus, cytosol, and mitochondria after treating neurons with palmitate-BSA for 24 h, 48 h, and 72 h. The expressions were analyzed by western blotting. The target protein expressions relative to GAPDH expression were displayed on the right. The experiments were performed in triplicate, and each value represents mean ± SD. ^∗^*P* < 0.05; ^∗∗∗^*P* < 0.001. (b) The distribution of TXNIP in neurons after treating with palmitate-BSA for 24 h, 48 h, and 72 h, which was detected under the fluorescence microscope. (c) The interaction of TXNIP with TRX1-Cyto, TRX2-Mito, and NLRP3-Cyto, the interaction of TRX1 with ASK1-Cyto, and the interaction of TRX2 with ASK1-Mito were determined by co-IP analysis after treating with palmitate-BSA for 24 h, 48 h, and 72 h. The target protein expressions relative to input were displayed below. The experiments were performed in triplicate, and each value represents mean ± SD. ^∗∗^*P* < 0.01; ^∗∗∗^*P* < 0.001.

TXNIP-mediated oxidative stress and apoptosis are known to be involved in their interaction with TRX1/2 and NLRP3. TRX-1 and NLRP3 are mainly located in the cytoplasm and TRX-2 in the mitochondria. We next investigated the distribution and changes of TXNIP in DRG neurons treated with palmitate. As shown in Figure 6(a), TXNIP was transferred from the nucleus into the cytosol and mitochondria after treating with palmitate for 24 h and 48 h, and then, the majority of TXNIP was accumulated in cytosol at 72 h after the treatment (*P* < 0.05 or 0.001). In line with the western blotting analysis, TXNIP stained in red was accumulated in cytosol along with the time (Figure 6(b)).

Since TXNIP interacting with TRX1, TRX2, and NLRP3 is required for increased oxidative stress, apoptosis, and inflammation, we next performed co-IP assay to examine changes of TXNIP-TRX1, TXNIP-TRX2, and TXNIP-NLRP3 complexes in DRG neurons treated with palmitate. Our results demonstrated that the level of TXNIP-TRX1 and TXNIP-NLRP3 complexes in cytosol was significantly increased over time in palmitate-treated neurons (*P* < 0.05 or 0.001), and the level of TXNIP-TRX2 complex in mitochondria was also increased within 48 h treatment (*P* < 0.001) while the level of this complex was decreased after 72 h treatment of palmitate (*P* < 0.05) (Figure 6(c), left). Our findings are thus consistent with the previous results suggesting the mitochondrial shuttling of TXNIP.

ASK1, a redox-regulated apoptosis signal kinase, is usually bound to TRX2 under basal conditions. However, in the condition of stress or TXNIP translocation to the mitochondria, ASK1-TRX1 binding and ASK1-TRX2 binding will be disrupted, triggering an apoptotic signal cascade. We thus explored changes of ASK1-TRX1 and ASK1-TRX2 complexes. The results demonstrated that ASK1-TRX1 and ASK1-TRX2 complexes were markedly departed by TXNIP introduction (*P* < 0.01 or 0.001) (Figure 6(c), right).

## 4. Discussion

Verapamil, a known inhibitor of calcium channels, has been widely used as an antihypertensive for more than 30 years. Recently, a phase 2 clinical trial showed that verapamil use might be a safe and effective novel approach to delay the progression of type 1 diabetes [34]. However, no study has been performed to determine whether verapamil use has beneficial effects on the onset and progression of neuropathy in prediabetes or recent-onset diabetes with dyslipidemia. In a novel set of experiments, our data showed that oral use of verapamil remarkably improved prediabetic neuropathy in HFD-fed mice, as reflected by significantly increased MNCV and SNCV, suggesting that verapamil may have a therapeutic effect on the clinical treatment of prediabetic neuropathy.

**Figure 7:**
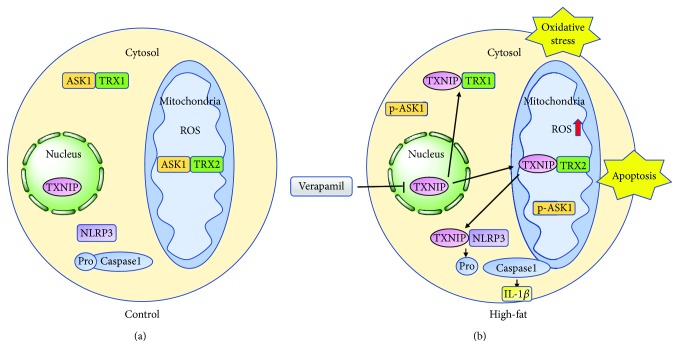
The postulated mechanism of verapamil attenuates the inflammation and apoptosis of neurons induced by palmitate through TXNIP inhibition. (a) Under normal conditions: TXNIP rests in the nucleus. TRX1-ASK1 and TRX2-ASK1 binding in the cytosol and mitochondria maintains low ROS. (b) Under high-fat conditions: verapamil inhibits the expression of TXNIP, which shuttles to the cytosol and mitochondria, binds to TRX1 and TRX2, and then transfers to cytosol to combine NLPR3, eventually inhibiting the oxidative stress and apoptosis induced by palmitate treatment.

In this study, we provided the evidence that TXNIP upregulation is associated with the development of prediabetic neuropathy in the mice fed with an HFD. In terms of mechanism, increased TXNIP in dorsal root ganglion with abnormal lipid deposition is transferred into the cytoplasm and shuttled to mitochondria. In cytoplasm, TXNIP binding to TRX1 results in the increased oxidative stress and inflammation. In mitochondria, TXNIP binding to TRX2 induced dysfunction of mitochondria and apoptosis cascade reaction. TXNIP isolated from TRX2 then shuttles to cytoplasm and binds to NLRP3, resulting increased TXNIP-NLRP3 complex, which induced the release of IL-1*β* and the development of inflammation. Thus, apoptosis and inflammation of dorsal root ganglion neuron eventually cause the neural dysfunction. Furthermore, we showed for the first time that verapamil as an inhibitor of TXNIP expression improved prediabetic neuropathy in the HFD-fed mice. This process is shown in Figure 7.

Our finding suggests that TXNIP might be a potential target for the treatment of neuropathy in prediabetic patients with dyslipidemia.

Hyperglycemia is clearly shown to contribute to the peripheral nerve injury through increasing OS and AGE. However, efficacy of hypoglycemic therapy or antioxidant treatment is very limited in delaying the progression of DN. Recently, an emerging idea is that dyslipidemia is also a key factor leading to the development of DN [6, 35, 36]. Consistent with this, in our previous study, the mice fed with an HFD showed deficits in MNCV and SNCV, thermal hyperalgesia, reduced mean dendrite length, and VEGF-A expression in the plantar skin and increased 12/15-LOX in the sciatic nerve. Therefore, the mice fed with an HFD were used to study the development and progression of prediabetic neuropathy.

By 34 weeks, the mice fed with an HFD showed increased level of serum TG, TC, HDL-C, and LDL-C. Similarly, verapamil significantly decreased TG, TC, HDL-C, and LDL-C concentration, which is basically consistent with the results in the two previous studies showing that verapamil significantly decreased TG, TC, or LDL-C [37, 38]. We also observed decreased MNCV and SNCV in the mice fed with an HFD, suggesting that the neuropathy appeared. This is consistent with the previous study showing the emergence of neuropathy after 34 weeks on a high-fat diet [6]. Of note, verapamil partially restores decreased nerve conduction velocity. These findings show that verapamil-mediated improvement of neuropathy might be associated with improvement of dyslipidemia.

Following 34 weeks, we observed that mice fed with an HFD displayed impaired glucose tolerance, as reflected by the ipGTT. It should be noted that the ipGTT in the present study was imperfect considering lack of a 15-minute test point. However, the AUC of blood glucose level in mice fed with an HFD is higher than that of normal mice. This trend is consistent with the study of Vincent et al. in which the ipGTT was tested at 0, 5, 15, 30, 60, and 120 minutes. In addition, a significant increase in plasma insulin levels and an increase in GHb were observed in the study of Vincent et al. These increases were small compared to typical 2 diabetic mice with much higher level of the plasma insulin and GHb, but the extent of neuropathy is comparable [6]. In the clinical trial, similar data were observed that dyslipidemia, not hyperglycemia, was more closely associated with neuropathy progression in 427 trial participants [39]. This suggests that dyslipidemia plays an important role in the onset of neuropathy and dyslipidemia-induced neuropathy might precede the development of diabetes. However, the molecular mechanism of dyslipidemia-induced neuropathy is unclear. In the present study, verapamil, identified as an inhibitor of TXNIP, improved dyslipidemia-induced neuropathy, which provides a clue to clarify this potential mechanism.

We know that the expression of TXNIP, an endogenous inhibitor of TRX, is regulated by glucose [40] and free fatty acid [41]. Recently, TXNIP is shown to be associated with the vascular complications of diabetes. This role for TXNIP in the development of diabetic retinopathy [42] and nephropathy has been shown to be involved in NLRP3 inflammasome activation and release of IL-1*β* [43]. Based on the findings, we proposed that diabetic neuropathy induced by HFD was likely to be associated with TXNIP upregulation. In the previous study, TXNIP was found to be increased in the DRG of STZ-induced diabetic mice [22]. However, they did not provide the functional evidence that TXNIP upregulation is associated with diabetic neuropathy. In the present study, we confirmed through TXNIP knockdown that its upregulation contributed to the development of prediabetic neuropathy in the HFD-fed mice, as reflected by the increased MNCV and SNCV. Moreover, we showed that TXNIP upregulation induced by palmitate, a compound which can lead to triglyceride accumulation in cells, induced the apoptosis of DRG neurons *in vitro*. On the other hand, TXNIP inhibition caused by siRNA-TXNIP or TXNIP inhibitor verapamil significantly attenuated the apoptosis of DRG neurons treated with palmitate. Thus, our findings suggest that onset and progression of diabetic neuropathy might be linked to the increased expression of TXNIP. It is known that the primary role of TXNIP is to inhibit the function of TRX, an important redox protein that controls levels of ROS in cells and limits damage from oxidative stress. Cytoplasmic thioredoxin (TRX1)-ASK1 complex is linked to regulation of ROS. Mitochondrial thioredoxin (TRX2)-ASK1 complex, like cytoplasmic TRX1, regulates oxidative stress in mitochondria. Once TXNIP shuttles to the cytosol and mitochondria, TRX1-ASK1 and TRX2-ASK2 are disrupted, respectively [11, 44]. In the present study, we observed disassociation of TRX1-ASK1/ASK2 and association of TXNIP-TRX1/TRX2 in the DRG neurons after palmitate treatment for 24 h and 48 h. Interestingly, in the DRG neurons after 72 h of palmitate treatment, we observed disassociation of TXNIP-TRX2 complex, which resulted from continuously increased ROS and mitochondria-mediated apoptosis. We accordingly observed the increased apoptosis in DRG neurons treated with palmitate.

Studies showed that TXNIP from mitochondria shuttles to the cytoplasm where it binds to NLRP3, which caused the activation of NLRP3 inflammasome, triggering caspase-1 activation leading to processing of interleukin-1*β* (IL-1*β*) [45]. Consistent with this, we observed significantly increased levels of TXNIP-NLRP3 complex in the DRG neurons 72 h after palmitate treatment.

Our study also showed that siRNA-mediated knockdown of TXNIP attenuated DN in HFD-fed mice, as reflected by increased MNCV and SNCV. In cellular level, we also found that TXNIP knockdown attenuated the apoptosis of DRG neurons treated with palmitate. Moreover, these changes were all associated with inhibition of NLRP3, caspase-1, and IL-1*β*. These findings thus provided a mechanistic explanation of the neuronal apoptosis caused by hyperlipidemia or palmitate.

Our results thus suggest that TXNIP might be a potential target for the treatment of prediabetic neuropathy. In fact, we observed that pharmacological inhibition of TXNIP caused by verapamil, an inhibitor of TXNIP, increased MNCV and SNCV in HFD-fed mice, suggesting that verapamil improved diabetic neuropathy. Similar with TXNIP knockdown, verapamil-mediated inhibition of TXNIP dose-dependently decreased the apoptosis of neurons treated with palmitate. Therefore, our finding suggests that verapamil, a traditional antihypertensive and antiarrhythmic, is likely to be effective in treating or delaying neuropathy of prediabetic patients especially with dyslipidemia.

## Data Availability

The data used to support the findings of this study are included within the article.
